# Tetra­kis[diamminesilver(I)] bis­(2-hy­droxy-5-methyl­benzene-1,3-disulfonate) monohydrate

**DOI:** 10.1107/S1600536811048124

**Published:** 2011-11-19

**Authors:** Li-Wei Zhang, Shan Gao, Seik Weng Ng

**Affiliations:** a Key Laboratory of Functional Inorganic Material Chemistry, Ministry of Education, Heilongjiang University, Harbin 150080, People’s Republic of China; b Department of Chemistry, University of Malaya, 50603 Kuala Lumpur, Malaysia; c Chemistry Department, Faculty of Science, King Abdulaziz University, PO Box 80203 Jeddah, Saudi Arabia

## Abstract

In the crystal structure of the title salt, [Ag(NH_3_)_2_]_4_(C_7_H_6_O_7_S_2_)_2_·H_2_O, the four independent Ag^I^ complex cations all lie on special positions of *m* site symmetry, as do the two independent 2-hy­droxy-5-methyl­benzene-1,3-disulfonate anions. The Ag^I^ cations exist in an almost linear coordination geometry [N—Ag—N = 175.2 (2), 178.08 (16), 175.8 (2) and 178.20 (19)°]. The water mol­ecule is disordered about a mirror plane. Two independent complex cations are linked by an Ag⋯Ag inter­action of 3.3151 (1) Å, furnishing a linear [Ag(NH_3_)_2_]_*n*_ polycationic chain running along *b*. The free complex cations, polycationic chain and 2-hy­droxy-5-methyl­benzene-1,3-disulfonate anions inter­act *via* N—H⋯O and O—H⋯O hydrogen bonds, forming a three-dimensional network.

## Related literature

For background literature, see: Deng *et al.* (2011 ▶). For the synthesis of disulfonic acid, see: Lambrechts *et al.* (1985 ▶). 
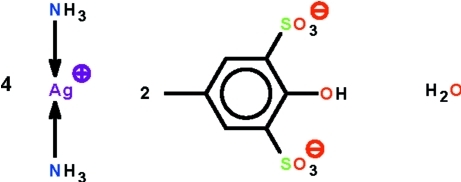


## Experimental

### 

#### Crystal data

[Ag(NH_3_)_2_]_4_(C_7_H_6_O_7_S_2_)_2_·H_2_O
*M* *_r_* = 1118.24
Monoclinic, 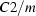

*a* = 21.6379 (8) Å
*b* = 6.5889 (2) Å
*c* = 24.7793 (8) Å
β = 108.015 (1)°
*V* = 3359.59 (19) Å^3^
*Z* = 4
Mo *K*α radiation
μ = 2.62 mm^−1^
*T* = 293 K
0.19 × 0.13 × 0.11 mm

#### Data collection

Rigaku RAXIS-RAPID IP diffractometer
Absorption correction: multi-scan (*ABSCOR*; Higashi, 1995 ▶) *T*_min_ = 0.636, *T*_max_ = 0.762
16616 measured reflections
4169 independent reflections
3613 reflections with *I* > 2σ(*I*)
*R*_int_ = 0.028

#### Refinement

*R*[*F* ^2^ > 2σ(*F* ^2^)] = 0.037
*wR*(*F* ^2^) = 0.104
*S* = 1.05
4169 reflections
274 parameters
30 restraints
H-atom parameters constrained
Δρ_max_ = 1.48 e Å^−3^
Δρ_min_ = −1.14 e Å^−3^

### 

Data collection: *RAPID-AUTO* (Rigaku, 1998 ▶); cell refinement: *RAPID-AUTO*; data reduction: *CrystalClear* (Rigaku/MSC, 2002 ▶); program(s) used to solve structure: *SHELXS97* (Sheldrick, 2008 ▶); program(s) used to refine structure: *SHELXL97* (Sheldrick, 2008 ▶); molecular graphics: *X-SEED* (Barbour, 2001 ▶); software used to prepare material for publication: *publCIF* (Westrip, 2010 ▶).

## Supplementary Material

Crystal structure: contains datablock(s) global, I. DOI: 10.1107/S1600536811048124/xu5387sup1.cif

Structure factors: contains datablock(s) I. DOI: 10.1107/S1600536811048124/xu5387Isup2.hkl

Additional supplementary materials:  crystallographic information; 3D view; checkCIF report

## Figures and Tables

**Table 1:** Hydrogen-bond geometry (Å, °)

*D*—H⋯*A*	*D*—H	H⋯*A*	*D*⋯*A*	*D*—H⋯*A*
N1—H12⋯O10	0.88	2.12	2.977 (4)	166
N2—H21⋯O1*w*	0.88	2.20	2.955 (9)	143
N7—H72⋯O10^i^	0.88	2.33	3.135 (5)	152
N8—H82⋯O4^ii^	0.88	2.21	3.064 (4)	164
O3—H3⋯O2	0.84	1.90	2.582 (5)	138
O9—H9⋯O7	0.84	1.95	2.612 (6)	134
O1*w*—H1*w*1⋯O11^iii^	0.84	1.91	2.720 (8)	160
O1*w*—H1*w*2⋯O6^iv^	0.84	1.94	2.762 (11)	166
O1*w*—H1*w*2⋯O8^v^	0.84	1.94	2.716 (11)	153

Symmetry codes: (i) 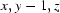; (ii) 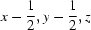; (iii) 
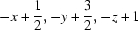
; (iv) 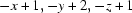; (v) 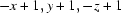.

**Fig. 1.**
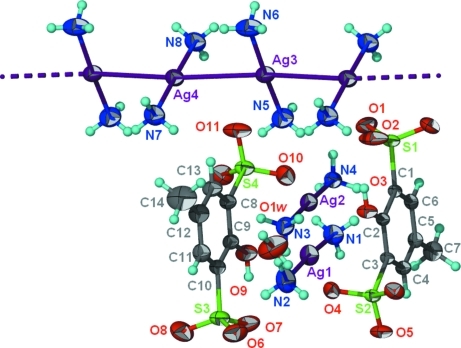
Thermal ellipsoid plot (Barbour, 2001) of 4[Ag(NH3)2]+(C7H6O7S2)22-.H2O at the 50% probability level; hydrogen atoms are drawn as spheres of arbitrary radius. Symmetry-related atoms are not labeled.

## supplementary crystallographic information

### Comment

The silver derivative of hydroxy-5-methylbenzene-1,3-disulfonic acid as well as that of other *o*-hydroxy arenesulfonic acids are coordination polymers that exhibit luminescence; these feature silver–sulfonate covalent bonds (Deng *et al.*, 2011). A variation of the synthesis yielded the title salt (Scheme I) in which the sulfonate dianion interacts with the metal atom indirectly, in an outer-sphere type of coordination. The Ag^I^ atoms in the salt, 2[Ag(NH_3_)_2_]^+^ (C_7_H_6_O_7_S_2_)^2-.^0.5H_2_O, exist in a linear coordination geometry. The four independent cations all lie on mirror planes, as do the two independent anions. The lattice water molecule is disordered about a mirror plane (Fig. 1). Two independent cations are linked by an Ag···Ag interaction of 3.3151 (1) Å to furnish a linear polycation [Ag(NH_3_)_2_]*_n_* chain running along *b*. The free cations, polycationic chain and anions interact by N–H···O and O–H···O hydrogen bonds to form a three-dimensional network (Table 1).

### Experimental

Silver nitrate (2 mmol) and 2-hydroxy-5-methylbenzene-1,3-disulfonic acid (1 mmol) were mixed in water (15 ml); the pH value was adjusted to *ca* 6 by the addition of ammonium hydroxide. The solution was filtered; colorless crystals were isolated from the solution, which was kept away from light, after several days.

### Refinement

Carbon-bound H-atoms were generated geometrically and were included in the riding model approximation for the aromatic ones only; the methyl ones were placed in calculated positions [C–H 0.93–0.98 Å, *U*(H) 1.2–1.5*U*_eq_(C)]. The amino and water H-atoms were similarly placed [N–H 0.88 and O–H 0.84 Å, *U*(H) 1.2–1.5*U*_eq_(N,O)].

The O atoms of one –SO_3_ groups were allowed to refine off the mirror plane. The S–O distances were restrained to within ±0.01 Å of each other, as were the O···O distances. Their anisotropic temperature factors were restrained to be nearly isotropic.

The largest peak was 0.92 Å from Ag1 and deepest hole 0.66 Å from Ag1.

Omitted because of bad disagreement were -1 1 1 and 1 1 2 reflections.

### Crystal data

**Table:**

4(AgH_6_N_2_^+^)·2(C_7_H_6_O_7_S_2_^2^^−^)·H_2_O	*F*(000) = 2200
*M**_r_* = 1118.24	*D*_x_ = 2.211 Mg m^−^^3^
Monoclinic, *C*2/*m*	Mo *K*α radiation, λ = 0.71073 Å
Hall symbol: -C 2y	Cell parameters from 13532 reflections
*a* = 21.6379 (8) Å	θ = 3.0–27.5°
*b* = 6.5889 (2) Å	µ = 2.62 mm^−^^1^
*c* = 24.7793 (8) Å	*T* = 293 K
β = 108.015 (1)°	Prism, colorless
*V* = 3359.59 (19) Å^3^	0.19 × 0.13 × 0.11 mm
*Z* = 4	

### Data collection

**Table:**

Rigaku RAXIS-RAPID IP diffractometer	4169 independent reflections
Radiation source: fine-focus sealed tube	3613 reflections with *I* > 2σ(*I*)
graphite	*R*_int_ = 0.028
ω scan	θ_max_ = 27.5°, θ_min_ = 3.0°
Absorption correction: multi-scan (*ABSCOR*; Higashi, 1995)	*h* = −28→28
*T*_min_ = 0.636, *T*_max_ = 0.762	*k* = −7→8
16616 measured reflections	*l* = −32→32

### Refinement

**Table:**

Refinement on *F*^2^	Primary atom site location: structure-invariant direct methods
Least-squares matrix: full	Secondary atom site location: difference Fourier map
*R*[*F*^2^ > 2σ(*F*^2^)] = 0.037	Hydrogen site location: inferred from neighbouring sites
*wR*(*F*^2^) = 0.104	H-atom parameters constrained
*S* = 1.05	*w* = 1/[σ^2^(*F*_o_^2^) + (0.0669*P*)^2^ + 3.482*P*] where *P* = (*F*_o_^2^ + 2*F*_c_^2^)/3
4169 reflections	(Δ/σ)_max_ = 0.001
274 parameters	Δρ_max_ = 1.48 e Å^−^^3^
30 restraints	Δρ_min_ = −1.14 e Å^−^^3^

### Fractional atomic coordinates and isotropic or equivalent isotropic displacement parameters (Å^2^)

**Table:**

	*x*	*y*	*z*	*U*_iso_*/*U*_eq_	Occ. (<1)
Ag1	0.34683 (2)	1.0000	0.380433 (17)	0.05373 (13)	
Ag2	0.15158 (2)	0.5000	0.079762 (18)	0.04880 (13)	
Ag3	0.006423 (19)	0.5000	0.239383 (18)	0.05088 (13)	
Ag4	0.017750 (18)	0.0000	0.254010 (17)	0.04835 (13)	
S1	0.05853 (5)	1.0000	0.09757 (5)	0.0374 (2)	
S2	0.32892 (5)	1.0000	0.15613 (4)	0.0338 (2)	
S3	0.45119 (6)	0.5000	0.38736 (6)	0.0533 (3)	
S4	0.18163 (6)	0.5000	0.30777 (5)	0.0452 (3)	
O1	0.02468 (13)	0.8174 (4)	0.07320 (13)	0.0606 (7)	
O2	0.07766 (17)	1.0000	0.15926 (15)	0.0723 (14)	
O3	0.20199 (15)	1.0000	0.17880 (12)	0.0414 (7)	
H3	0.1695	1.0000	0.1905	0.062*	
O4	0.33304 (11)	0.8183 (4)	0.18995 (11)	0.0487 (6)	
O5	0.37405 (16)	1.0000	0.12320 (15)	0.0514 (9)	
O6	0.4888 (4)	0.6529 (10)	0.4256 (3)	0.073 (2)	0.50
O7	0.4393 (3)	0.5594 (10)	0.3290 (2)	0.079 (3)	0.50
O8	0.4777 (5)	0.3031 (10)	0.4000 (3)	0.080 (3)	0.50
O9	0.31379 (17)	0.5000	0.29697 (14)	0.0512 (9)	
H9	0.3483	0.5000	0.2882	0.077*	
O10	0.18069 (14)	0.6822 (5)	0.27464 (12)	0.0616 (7)	
O11	0.13145 (19)	0.5000	0.33504 (19)	0.0742 (13)	
O1w	0.4062 (4)	1.1079 (14)	0.5739 (3)	0.105 (3)	0.50
H1w1	0.3988	1.1011	0.6053	0.157*	0.50
H1w2	0.4342	1.1975	0.5752	0.157*	0.50
N1	0.2833 (2)	1.0000	0.29685 (18)	0.0546 (11)	
H11	0.3060	1.0000	0.2729	0.082*	
H12	0.2587	0.8909	0.2913	0.082*	
N2	0.4171 (3)	1.0000	0.4614 (2)	0.0697 (14)	
H21	0.3975	1.0000	0.4877	0.104*	
H22	0.4415	1.1091	0.4652	0.104*	
N3	0.2209 (2)	0.5000	0.16227 (19)	0.0509 (10)	
H31	0.2604	0.5000	0.1593	0.076*	
H32	0.2155	0.3909	0.1808	0.076*	
N4	0.0841 (2)	0.5000	−0.0049 (2)	0.0495 (10)	
H41	0.0441	0.5000	−0.0032	0.074*	
H42	0.0903	0.3909	−0.0231	0.074*	
N5	0.0525 (3)	0.5000	0.1760 (2)	0.0629 (13)	
H51	0.0230	0.5000	0.1424	0.094*	
H52	0.0770	0.3909	0.1796	0.094*	
N6	−0.0463 (3)	0.5000	0.2980 (2)	0.0766 (17)	
H61	−0.0882	0.5000	0.2796	0.115*	
H62	−0.0364	0.6091	0.3194	0.115*	
N7	0.1036 (2)	0.0000	0.3228 (2)	0.0601 (12)	
H71	0.0938	0.0000	0.3548	0.090*	
H72	0.1265	−0.1091	0.3213	0.090*	
N8	−0.0663 (2)	0.0000	0.1835 (2)	0.0584 (11)	
H81	−0.0552	0.0000	0.1522	0.088*	
H82	−0.0894	0.1091	0.1843	0.088*	
C1	0.13312 (19)	1.0000	0.08124 (17)	0.0287 (8)	
C6	0.1288 (2)	1.0000	0.02360 (17)	0.0318 (8)	
H6	0.0881	1.0000	−0.0037	0.038*	
C5	0.1838 (2)	1.0000	0.00656 (17)	0.0338 (8)	
C4	0.2439 (2)	1.0000	0.04819 (18)	0.0337 (8)	
H4	0.2814	1.0000	0.0373	0.040*	
C3	0.24984 (18)	1.0000	0.10525 (17)	0.0287 (7)	
C2	0.19375 (18)	1.0000	0.12287 (16)	0.0267 (7)	
C7	0.1787 (3)	1.0000	−0.05577 (19)	0.0491 (12)	
H7D	0.1338	1.0000	−0.0783	0.074*	
H7E	0.1996	0.8810	−0.0643	0.074*	0.50
H7F	0.1996	1.1190	−0.0643	0.074*	0.50
C8	0.2568 (2)	0.5000	0.36432 (19)	0.0369 (9)	
C9	0.3165 (2)	0.5000	0.35254 (19)	0.0363 (9)	
C10	0.3735 (2)	0.5000	0.3979 (2)	0.0384 (9)	
C11	0.3718 (3)	0.5000	0.4540 (2)	0.0453 (11)	
H11A	0.4104	0.5000	0.4839	0.054*	
C12	0.3132 (3)	0.5000	0.4652 (2)	0.0504 (12)	
C13	0.2559 (3)	0.5000	0.4194 (2)	0.0473 (11)	
H13	0.2161	0.5000	0.4264	0.057*	
C14	0.3111 (4)	0.5000	0.5256 (2)	0.091 (3)	
H14A	0.3546	0.5000	0.5514	0.136*	
H14B	0.2886	0.3810	0.5320	0.136*	0.50
H14C	0.2886	0.6190	0.5320	0.136*	0.50

### Atomic displacement parameters (Å^2^)

**Table:**

	*U*^11^	*U*^22^	*U*^33^	*U*^12^	*U*^13^	*U*^23^
Ag1	0.0562 (3)	0.0543 (2)	0.0451 (2)	0.000	0.00751 (18)	0.000
Ag2	0.0520 (2)	0.0438 (2)	0.0534 (2)	0.000	0.02034 (18)	0.000
Ag3	0.0450 (2)	0.0570 (3)	0.0545 (2)	0.000	0.02104 (18)	0.000
Ag4	0.0376 (2)	0.0523 (2)	0.0505 (2)	0.000	0.00692 (16)	0.000
S1	0.0219 (5)	0.0500 (6)	0.0398 (5)	0.000	0.0086 (4)	0.000
S2	0.0215 (4)	0.0427 (5)	0.0343 (5)	0.000	0.0042 (4)	0.000
S3	0.0344 (6)	0.0615 (8)	0.0669 (8)	0.000	0.0199 (6)	0.000
S4	0.0327 (6)	0.0611 (7)	0.0408 (6)	0.000	0.0099 (5)	0.000
O1	0.0402 (14)	0.0579 (15)	0.085 (2)	−0.0170 (12)	0.0207 (13)	−0.0071 (15)
O2	0.0343 (19)	0.145 (5)	0.043 (2)	0.000	0.0202 (16)	0.000
O3	0.0281 (15)	0.071 (2)	0.0256 (13)	0.000	0.0088 (11)	0.000
O4	0.0351 (12)	0.0492 (13)	0.0538 (14)	0.0031 (10)	0.0019 (10)	0.0137 (12)
O5	0.0266 (16)	0.080 (2)	0.0496 (18)	0.000	0.0147 (14)	0.000
O6	0.047 (4)	0.082 (5)	0.089 (5)	−0.019 (4)	0.018 (4)	−0.008 (4)
O7	0.054 (3)	0.121 (7)	0.071 (3)	−0.008 (3)	0.035 (3)	0.015 (4)
O8	0.070 (5)	0.064 (4)	0.116 (6)	0.017 (4)	0.044 (5)	−0.010 (4)
O9	0.046 (2)	0.077 (2)	0.0361 (16)	0.000	0.0204 (15)	0.000
O10	0.0518 (16)	0.0666 (17)	0.0573 (15)	0.0055 (14)	0.0038 (12)	0.0122 (14)
O11	0.0331 (19)	0.131 (4)	0.061 (2)	0.000	0.0171 (17)	0.000
O1w	0.087 (5)	0.143 (6)	0.087 (5)	−0.028 (4)	0.033 (4)	0.007 (4)
N1	0.051 (3)	0.065 (3)	0.040 (2)	0.000	0.0036 (18)	0.000
N2	0.076 (4)	0.058 (3)	0.059 (3)	0.000	−0.002 (3)	0.000
N3	0.058 (3)	0.049 (2)	0.051 (2)	0.000	0.024 (2)	0.000
N4	0.038 (2)	0.043 (2)	0.067 (3)	0.000	0.014 (2)	0.000
N5	0.067 (3)	0.073 (3)	0.055 (3)	0.000	0.029 (2)	0.000
N6	0.051 (3)	0.123 (5)	0.061 (3)	0.000	0.024 (2)	0.000
N7	0.053 (3)	0.067 (3)	0.051 (3)	0.000	0.003 (2)	0.000
N8	0.043 (2)	0.068 (3)	0.054 (3)	0.000	0.000 (2)	0.000
C1	0.0235 (18)	0.0303 (18)	0.0313 (18)	0.000	0.0071 (14)	0.000
C6	0.031 (2)	0.0316 (19)	0.0271 (18)	0.000	0.0002 (15)	0.000
C5	0.035 (2)	0.036 (2)	0.0281 (18)	0.000	0.0058 (16)	0.000
C4	0.032 (2)	0.037 (2)	0.034 (2)	0.000	0.0132 (16)	0.000
C3	0.0203 (17)	0.0318 (18)	0.0315 (18)	0.000	0.0046 (14)	0.000
C2	0.0255 (18)	0.0287 (17)	0.0255 (17)	0.000	0.0073 (14)	0.000
C7	0.057 (3)	0.061 (3)	0.030 (2)	0.000	0.014 (2)	0.000
C8	0.034 (2)	0.044 (2)	0.035 (2)	0.000	0.0126 (17)	0.000
C9	0.037 (2)	0.039 (2)	0.036 (2)	0.000	0.0157 (18)	0.000
C10	0.032 (2)	0.039 (2)	0.046 (2)	0.000	0.0137 (19)	0.000
C11	0.044 (3)	0.049 (3)	0.039 (2)	0.000	0.006 (2)	0.000
C12	0.052 (3)	0.068 (3)	0.033 (2)	0.000	0.014 (2)	0.000
C13	0.044 (3)	0.062 (3)	0.041 (2)	0.000	0.021 (2)	0.000
C14	0.091 (5)	0.150 (8)	0.033 (3)	0.000	0.023 (3)	0.000

### Geometric parameters (Å, °)

**Table:**

Ag3—Ag4	3.3151 (1)	N2—H22	0.8800
Ag1—N1	2.102 (4)	N3—H31	0.8800
Ag1—N2	2.110 (5)	N3—H32	0.8800
Ag2—N3	2.129 (5)	N4—H41	0.8800
Ag2—N4	2.154 (5)	N4—H42	0.8800
Ag3—N5	2.105 (4)	N5—H51	0.8800
Ag3—N6	2.107 (5)	N5—H52	0.8800
Ag4—N8	2.096 (4)	N6—H61	0.8800
Ag4—N7	2.097 (5)	N6—H62	0.8800
S1—O1	1.441 (3)	N7—H71	0.8800
S1—O1^i^	1.441 (3)	N7—H72	0.8800
S1—O2	1.455 (4)	N8—H81	0.8800
S1—C1	1.781 (4)	N8—H82	0.8800
S2—O4^i^	1.448 (2)	C1—C2	1.396 (5)
S2—O4	1.448 (2)	C1—C6	1.402 (6)
S2—O5	1.453 (3)	C6—C5	1.380 (6)
S2—C3	1.784 (4)	C6—H6	0.9300
S3—O8^ii^	1.414 (6)	C5—C4	1.388 (6)
S3—O8	1.414 (6)	C5—C7	1.514 (6)
S3—O7	1.442 (5)	C4—C3	1.380 (6)
S3—O7^ii^	1.442 (5)	C4—H4	0.9300
S3—O6	1.448 (6)	C3—C2	1.411 (5)
S3—O6^ii^	1.448 (5)	C7—H7D	0.9600
S3—C10	1.778 (5)	C7—H7E	0.9600
S4—O11	1.446 (4)	C7—H7F	0.9600
S4—O10^ii^	1.451 (3)	C8—C13	1.370 (6)
S4—O10	1.451 (3)	C8—C9	1.408 (6)
S4—C8	1.789 (5)	C9—C10	1.389 (7)
O3—C2	1.342 (5)	C10—C11	1.402 (7)
O3—H3	0.8400	C11—C12	1.379 (7)
O9—C9	1.360 (5)	C11—H11A	0.9300
O9—H9	0.8400	C12—C13	1.398 (7)
O1w—H1w1	0.8430	C12—C14	1.514 (7)
O1w—H1w2	0.8400	C13—H13	0.9300
N1—H11	0.8800	C14—H14A	0.9600
N1—H12	0.8800	C14—H14B	0.9600
N2—H21	0.8800	C14—H14C	0.9600
			
N1—Ag1—N2	175.2 (2)	Ag3—N6—H62	109.5
N3—Ag2—N4	178.08 (16)	H61—N6—H62	109.5
N5—Ag3—N6	175.8 (2)	Ag4—N7—H71	109.5
N5—Ag3—Ag4^iii^	92.607 (18)	Ag4—N7—H72	109.5
N6—Ag3—Ag4^iii^	87.830 (19)	H71—N7—H72	109.5
N5—Ag3—Ag4	92.607 (18)	Ag4—N8—H81	109.5
N6—Ag3—Ag4	87.830 (19)	Ag4—N8—H82	109.5
Ag4^iii^—Ag3—Ag4	167.20 (2)	H81—N8—H82	109.5
N8—Ag4—N7	178.20 (19)	C2—C1—C6	120.2 (4)
N8—Ag4—Ag3^iv^	83.864 (11)	C2—C1—S1	122.9 (3)
N7—Ag4—Ag3^iv^	96.076 (11)	C6—C1—S1	116.9 (3)
N8—Ag4—Ag3	83.864 (11)	C5—C6—C1	121.3 (4)
N7—Ag4—Ag3	96.076 (11)	C5—C6—H6	119.3
Ag3^iv^—Ag4—Ag3	167.20 (2)	C1—C6—H6	119.3
O1—S1—O1^i^	113.3 (3)	C6—C5—C4	118.1 (4)
O1—S1—O2	112.52 (15)	C6—C5—C7	121.0 (4)
O1^i^—S1—O2	112.52 (15)	C4—C5—C7	120.9 (4)
O1—S1—C1	106.48 (13)	C3—C4—C5	122.0 (4)
O1^i^—S1—C1	106.48 (13)	C3—C4—H4	119.0
O2—S1—C1	104.8 (2)	C5—C4—H4	119.0
O4^i^—S2—O4	111.5 (2)	C4—C3—C2	120.1 (4)
O4^i^—S2—O5	113.14 (13)	C4—C3—S2	119.3 (3)
O4—S2—O5	113.14 (13)	C2—C3—S2	120.7 (3)
O4^i^—S2—C3	106.43 (12)	O3—C2—C1	123.9 (3)
O4—S2—C3	106.43 (12)	O3—C2—C3	117.9 (3)
O5—S2—C3	105.5 (2)	C1—C2—C3	118.2 (3)
O8—S3—O7	114.0 (4)	C5—C7—H7D	109.5
O8—S3—O6	112.7 (4)	C5—C7—H7E	109.5
O7—S3—O6	110.9 (4)	H7D—C7—H7E	109.5
O8—S3—C10	107.7 (5)	C5—C7—H7F	109.5
O7—S3—C10	105.2 (3)	H7D—C7—H7F	109.5
O6—S3—C10	105.7 (4)	H7E—C7—H7F	109.5
O11—S4—O10^ii^	112.54 (15)	C13—C8—C9	120.2 (4)
O11—S4—O10	112.54 (15)	C13—C8—S4	119.4 (4)
O10^ii^—S4—O10	111.7 (3)	C9—C8—S4	120.5 (3)
O11—S4—C8	105.5 (2)	O9—C9—C10	124.6 (4)
O10^ii^—S4—C8	107.06 (14)	O9—C9—C8	117.1 (4)
O10—S4—C8	107.06 (14)	C10—C9—C8	118.4 (4)
C2—O3—H3	120.0	C9—C10—C11	120.8 (4)
C9—O9—H9	120.0	C9—C10—S3	121.7 (4)
H1w1—O1w—H1w2	110.0	C11—C10—S3	117.5 (4)
Ag1—N1—H11	109.5	C12—C11—C10	120.5 (5)
Ag1—N1—H12	109.5	C12—C11—H11A	119.7
H11—N1—H12	109.5	C10—C11—H11A	119.7
Ag1—N2—H21	109.5	C11—C12—C13	118.4 (4)
Ag1—N2—H22	109.5	C11—C12—C14	120.7 (5)
H21—N2—H22	109.5	C13—C12—C14	120.8 (5)
Ag2—N3—H31	109.5	C8—C13—C12	121.7 (4)
Ag2—N3—H32	109.5	C8—C13—H13	119.1
H31—N3—H32	109.5	C12—C13—H13	119.1
Ag2—N4—H41	109.5	C12—C14—H14A	109.5
Ag2—N4—H42	109.5	C12—C14—H14B	109.5
H41—N4—H42	109.5	H14A—C14—H14B	109.5
Ag3—N5—H51	109.5	C12—C14—H14C	109.5
Ag3—N5—H52	109.5	H14A—C14—H14C	109.5
H51—N5—H52	109.5	H14B—C14—H14C	109.5
Ag3—N6—H61	109.5		
			
O1—S1—C1—C2	−119.42 (14)	O11—S4—C8—C9	180.0
O1^i^—S1—C1—C2	119.42 (14)	O10^ii^—S4—C8—C9	59.94 (14)
O2—S1—C1—C2	0.0	O10—S4—C8—C9	−59.94 (14)
O1—S1—C1—C6	60.58 (14)	C13—C8—C9—O9	180.0
O1^i^—S1—C1—C6	−60.58 (14)	S4—C8—C9—O9	0.0
O2—S1—C1—C6	180.0	C13—C8—C9—C10	0.000 (1)
C2—C1—C6—C5	0.0	S4—C8—C9—C10	180.0
S1—C1—C6—C5	180.0	O9—C9—C10—C11	180.000 (1)
C1—C6—C5—C4	0.0	C8—C9—C10—C11	0.000 (1)
C1—C6—C5—C7	180.0	O9—C9—C10—S3	0.0
C6—C5—C4—C3	0.0	C8—C9—C10—S3	180.0
C7—C5—C4—C3	180.0	O8^ii^—S3—C10—C9	105.6 (3)
C5—C4—C3—C2	0.0	O8—S3—C10—C9	−105.6 (3)
C5—C4—C3—S2	180.0	O7—S3—C10—C9	16.3 (3)
O4^i^—S2—C3—C4	120.47 (12)	O7^ii^—S3—C10—C9	−16.3 (3)
O4—S2—C3—C4	−120.47 (12)	O6—S3—C10—C9	133.8 (3)
O5—S2—C3—C4	0.0	O6^ii^—S3—C10—C9	−133.8 (3)
O4^i^—S2—C3—C2	−59.53 (12)	O8^ii^—S3—C10—C11	−74.4 (3)
O4—S2—C3—C2	59.53 (12)	O8—S3—C10—C11	74.4 (3)
O5—S2—C3—C2	180.0	O7—S3—C10—C11	−163.7 (3)
C6—C1—C2—O3	180.0	O7^ii^—S3—C10—C11	163.7 (3)
S1—C1—C2—O3	0.0	O6—S3—C10—C11	−46.2 (3)
C6—C1—C2—C3	0.0	O6^ii^—S3—C10—C11	46.2 (3)
S1—C1—C2—C3	180.0	C9—C10—C11—C12	0.000 (1)
C4—C3—C2—O3	180.0	S3—C10—C11—C12	180.000 (1)
S2—C3—C2—O3	0.0	C10—C11—C12—C13	0.000 (1)
C4—C3—C2—C1	0.0	C10—C11—C12—C14	180.000 (2)
S2—C3—C2—C1	180.0	C9—C8—C13—C12	0.0
O11—S4—C8—C13	0.0	S4—C8—C13—C12	180.0
O10^ii^—S4—C8—C13	−120.06 (14)	C11—C12—C13—C8	0.000 (1)
O10—S4—C8—C13	120.06 (14)	C14—C12—C13—C8	180.000 (1)

Symmetry codes: (i) *x*, −*y*+2, *z*; (ii) *x*, −*y*+1, *z*; (iii) *x*, *y*+1, *z*; (iv) *x*, *y*−1, *z*.

### Hydrogen-bond geometry (Å, °)

**Table:**

*D*—H···*A*	*D*—H	H···*A*	*D*···*A*	*D*—H···*A*
N1—H12···O10	0.88	2.12	2.977 (4)	166
N2—H21···O1w	0.88	2.20	2.955 (9)	143
N7—H72···O10^iv^	0.88	2.33	3.135 (5)	152
N8—H82···O4^v^	0.88	2.21	3.064 (4)	164
O3—H3···O2	0.84	1.90	2.582 (5)	138
O9—H9···O7	0.84	1.95	2.612 (6)	134
O1w—H1w1···O11^vi^	0.84	1.91	2.720 (8)	160
O1w—H1w2···O6^vii^	0.84	1.94	2.762 (11)	166
O1w—H1w2···O8^viii^	0.84	1.94	2.716 (11)	153

Symmetry codes: (iv) *x*, *y*−1, *z*; (v) *x*−1/2, *y*−1/2, *z*; (vi) −*x*+1/2, −*y*+3/2, −*z*+1; (vii) −*x*+1, −*y*+2, −*z*+1; (viii) −*x*+1, *y*+1, −*z*+1.
